# Quantitative Analysis of Protein and Gene Expression in Salivary Glands of Sjogren’s-Like Disease NOD Mice Treated by Bone Marrow Soup

**DOI:** 10.1371/journal.pone.0087158

**Published:** 2014-01-29

**Authors:** Kaori Misuno, Simon D. Tran, Saeed Khalili, Junwei Huang, Younan Liu, Shen Hu

**Affiliations:** 1 School of Dentistry, University of California Los Angeles, Los Angeles, California, United States of America; 2 Craniofacial Tissue Engineering and Stem Cells Laboratory, Faculty of Dentistry, McGill University, Montreal, Quebec, Canada; National Institutes of Health, United States of America

## Abstract

**Background:**

Bone marrow cell extract (termed as BM Soup) has been demonstrated to repair irradiated salivary glands (SGs) and restore saliva secretion in our previous study. In the present study, we aim to investigate if the function of damaged SGs in non-obese diabetic (NOD) mice can be restored by BM Soup treatment and the molecular alterations associated with the treatment.

**Methods:**

Whole BM cells were lysed and soluble intracellular contents (“BM Soup”) were injected I.V. into NOD mice. Tandem mass tagging with 2-D liquid chromatography-mass spectrometry was used to quantify proteins in the submandibular glands (SMGs) between untreated and BM Soup-treated mice. Quantitative PCR was used to identify genes with altered expression in the treated mice.

**Results BM Soup:**

restored salivary flow rates to normal levels and significantly reduced the focus scores of SMGs in NOD mice. More than 1800 proteins in SMG cells were quantified by the proteomic approach. Many SMG proteins involved in inflammation and apoptosis were found to be down-regulated whereas those involved in salivary gland biology and development/regeneration were up-regulated in the BM Soup-treated mice. qPCR analysis also revealed expression changes of growth factors and cytokines in the SMGs of the treated NOD mice.

**Conclusion:**

BM Soup treatment is effective to restore the function of damaged SGs in NOD mice. Through gene/protein expression analysis, we have found that BM Soup treatment might effectuate via inhibiting apoptosis, focal adhesion and inflammation whereas promoting development, regeneration and differentiation of the SG cells in NOD mice. These findings provide important insights on the potential mechanisms underlying the BM Soup treatment for functional restoration of damaged SGs in NOD mice. Additional studies are needed to further confirm the identified target genes and their related signaling pathways that are responsible for the BM Soup treatment.

## Introduction

Sjögren’s syndrome (SS) is a chronic autoimmune disease, with an estimated prevalence of ∼4 million in the US [1], [2]. Patients with SS suffer from dry mouth (xerostomia) and eyes (xeropthalmia) caused by lymphocytic infiltration of salivary and lachrymal glands. SS primarily affects women, with a ratio of 9∶1 over the occurrence in men. The disease is classified as primary SS when it exists by itself or secondary SS when it is associated with another autoimmune disease such as rheumatoid arthritis (RA), systemic lupus erythematosus (SLE) or systemic sclerosis. In addition, patients with SS have a significant higher risk of developing lymphoma than both healthy population and patients with other autoimmune diseases [3]. The most common form is mucosa-associated lymphoid tissue (MALT) lymphoma that remains localized in the affected salivary glands.

A typical pathological feature of SS is the presence of progressive lymphocytic infiltrates in the exocrine glands, such as salivary and lachrymal glands, where lymphocytes are not normally found [4]. Histopathological examination of the affected major salivary glands in patients with SS reveals a benign lymphoepithelial lesion, which is characterized by lymphocytic replacement of the salivary epithelium and accompanied by the formation of epimyoepithelial islands mainly composed of keratin-containing epithelial cells [5], [6]. The predominant cells in the salivary gland infiltrates are T cells, with a ratio of 3–5∶1 between CD4+ and CD8+ phenotypes. SS is also associated with B cell hyperactivity as manifested by the production of autoantibodies, hypergammaglobulinemia, formation of ectopic lymphoid structures within the inflamed tissues, and enhanced risk of B cell lymphoma [7]. B cells constitute approximately 20% of the total infiltrating population [6]. Various autoantibodies, such as anti-SS-A (Ro), anti-SS-B (La), anti-α-fodrin, anti-M3 muscarinic acetylcholine receptor (anti-M3R), anti-histone and anti-transglutamine are detected in serum or saliva fluids of patients with SS [4], [8]–[10].

The present level of mechanistic understanding of SS etiology and pathogenesis is inadequate, and accordingly there are no treatments that modify the evolution of SS in patients [11]. The efficacy of local treatments such as artificial tears or oral sprays is limited, whereas molecular-targeted therapies remain at early-stage clinical trials in SS patients. Recent studies suggest that cell-based therapies may emerge as a valid treatment approach for SS. For instance, transplantations with bone marrow-derived cells (BMDCs), splenic stem cells or salivary gland (adult) stem cells, have shown promises to restore the function of damaged salivary glands in animal models with SS-like disease or under irradiation treatment [12]–[14]. Transplantation of BMDCs is traditionally used for hematologic diseases, but there is an increasing interest in using BMDC treatments for non-hematologic disorders, including autoimmune diseases such as SS [12], [15]. Tran and colleagues initially observed that BMDCs from healthy male donors can differentiate into buccal (oral) epithelial cells of female transplant recipients and, recently, confirmed that a small percentage of Y-chromosome positive salivary cells (mean of <1%) are present in the biopsied salivary glands of five female patients, who received allogeneic BMDC or peripheral blood stem cell (PBSC) transplants from their brothers [16], [17]. The same research group has devoted significant efforts to demonstrating allogeneic BMDC transplants for treating SS in the NOD mice, which is an animal model displaying infiltrates of lymphocytes and a gradual loss of salivary function. In a recent study [18], they tested the long-term effects (52 weeks post-therapy) of complete Freund’s adjuvant (CFA, immunopotentiator) and MHC class I-matched normal BMDCs to 7-week old NOD mice prior to SS onset. At week 52 post-treatment, NOD mice treated with CFA+BMDCs were normoglycemic compared to the control group, and had their salivary glands’ function restored both quantitatively and qualitatively. Saliva production was significantly higher in BMDC-transplanted mice when compared to control NOD mice who continued to have their saliva production deteriorated over time. Similar strategy using BMDC transplants was also used to functionally restore irradiated salivary glands in animal models [19]–[21].

Recently, to test the mechanisms of action behind these reported successes using BMDCs for restoring salivary gland function, Tran and colleagues lysed whole BMDCs cells and injected the soluble intracellular contents (termed as “*Bone Marrow Soup*”; *BM Soup*) into mice with irradiation-injured SGs [22]. Their hypothesis was that if a paracrine effect was at play, *BM Soup* would protect salivary cells, increase tissue neovascularization, salivary function, and regeneration following irradiation damage. *BM Soup* was found as an efficient therapeutic agent as injections of live BMDCs. In addition, use of *BM Soup* is advantageous as it contains only the cell by-products and no whole live BMDCs which carry the risk of differentiating into unwanted/tumorigenic cell types in SGs.

Proteomic studies of SS have revealed a number of target proteins that may reflect the disease mechanisms or serve as diagnostic markers. It has been found that the proteomes of saliva or salivary gland cells from SS patients display increased expression of inflammatory proteins and decreased expression of acinar proteins [23]–[28]. We have discovered saliva protein and autoantibody biomarkers for SS using both mass spectrometry and protein microarray based proteomics. These markers have been successfully validated in independent patient cohorts, demonstrating their high sensitivity and specificity for the disease detection [9], [29], [30]. By using gene co-expression network analysis of expression microarray data and then correlation with proteomic analysis, we also revealed gene networks and signaling pathways that are associated with SS and associated MALT lymphoma [31].

The purpose of this study is to determine the molecular changes underlying the BM Soup treatment for restoration of damaged salivary glands in NOD mice. We report here a quantitative analysis of protein and gene expression changes in submandibular gland (SMG) tissue cells from untreated and BM Soup-treated NOD mice. Using proteomics and quantitative PCR, important target genes associated with BM Soup treatment have been identified and verified, which reveals potential molecular mechanisms underlying the functional restoration of damaged salivary glands in SS-like disease.

## Results

### Measurement of Salivary Gland Function of BM Soup Treated NOD Mice

**Figure 1:**
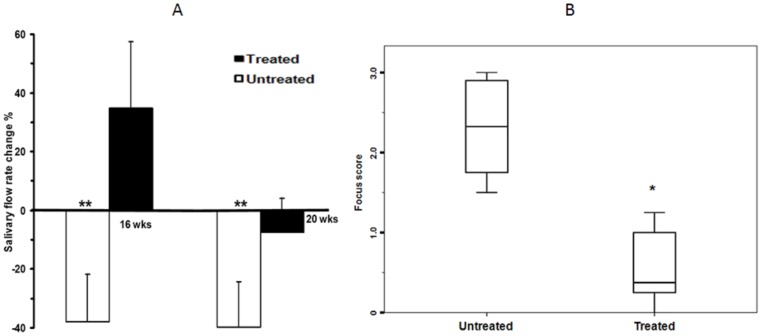
Salivary flow rates (SFRs, **Figure 1A**) and focus scores (**Figure 1B**) of the untreated and BM Soup-treated NOD mice (n = 6 mice per group). In Figure 1A, the white bars represent untreated NOD groups whereas the black bars represent treated NOD groups. **, p<0.01; *, p<0.05.

We assessed the efficacy of BM Soup in repairing SMGs that were damaged in NOD mice. At week 16, BM Soup-treated mice had a 35% improvement of their salivary flow rate (SFR) while untreated mice had a 38% reduction (**Fig. 1A**, p<0.05). At week 20, the SFR of BM Soup-treated mice was slightly reduced (−7%) whereas the SFR of untreated NOD mice further deteriorates to 40% below the baseline level. These data indicated that BM Soup treatment was valid in re-establishing saliva secretion. Meanwhile, the focus score of the SMGs in BM Soup-treated mice was significantly lower than that of the untreated NOD mice (**Fig. 1B**, p<0.05). The average focus scores were 2.34 in the untreated control group and 0.25 in the BM Soup-treated group, suggesting that BM Soup treatment suppresses the lymphocytic infiltration in the SMGs of the NOD mice.

### Quantitative Proteomic Analysis of the SMGs of BM Soup-treated NOD Mice

**Figure 2:**
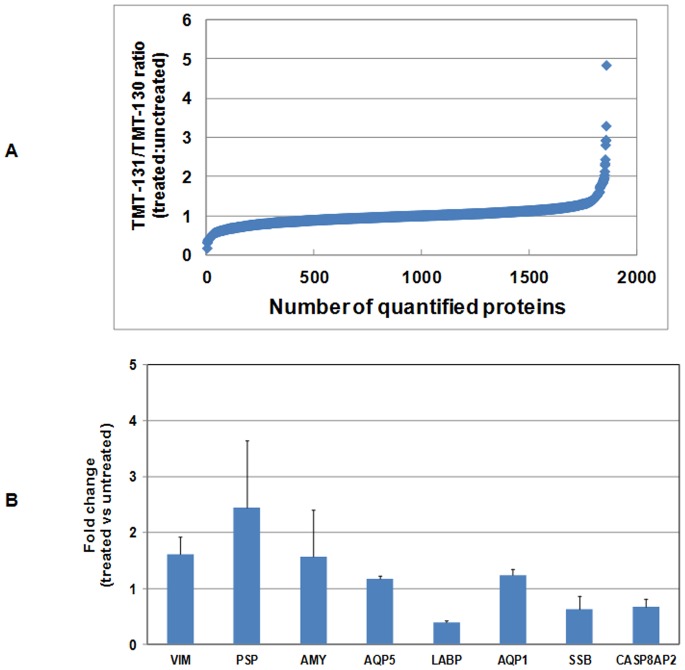
Quantitative proteomic analysis of SMG tissue proteins in untreated and BM SOUP treated NOD mice (n = 4 per group) using tandem mass tagging and 2-D LC-MS/MS. (A) Ratios of 131 to 130 represent the relative levels of proteins between BM SOUP-treated (TMT-131) and untreated (TMT-130) NOD mice. In total, 1885 proteins were identified with at least two matched, TMT-labeled peptides. (B) The relative expression levels of vimentin (VIM), parotid secretory component protein (PSP), alpha-amylase (AMY), aquaporin 5 (AQP5), lacrimal androgen-binding protein (LABP), Sjogren’s syndrome antigen B (SSB), aquaporin 1 (AQP1) and caspase 8 associated protein 2 (CASP8AP2) between untreated and BM Soup-treated mice.

In total, 1855 SMG tissue proteins were quantified between untreated and BM Soup-treated NOD mice based on TMT labeling and 2-D LC-MS/MS (**Fig. 2A**, Table S1). At least two unique peptides were confidently matched for each protein and also labeled with TMT. A representative list of proteins involved in inflammation, apoptosis or transcriptional regulation is shown in **Table 1**. These proteins were down-regulated in the SMG tissue cells of BM Soup-treated NOD mice when compared to untreated NOD mice. A second representative list of proteins involved in transcriptional regulation, stem cell biology or salivary gland biology is shown in **Table 2**. These SMG proteins were up-regulated in the BM Soup-treated NOD mice versus untreated NOD mice.

**Table 1:** A partial list of down-regulated SMG proteins in BM SOUP-treated NOD mice.

Accession Number	Ratio 131/130	# of Peptides	Protein Name
IPI00127754.1	0.399	2	Lacrimal androgen-binding protein
IPI00751076.1	0.405	17	Actin, cytoplasmic 2
IPI00120155.1	0.498	3	Interleukin-6 receptor beta chain precursor
IPI00126055.1	0.591	2	EGF-containing fibulin-like extracellular matrix protein 2 precursor
IPI00124675.1	0.622	2	Interferon-induced guanylate-binding protein 1
IPI00116861.4	0.625	2	Protein regulator of cytokinesis 1-like protein (Fragment)
IPI00221718.1	0.631	2	Polypeptide N-acetylgalactosaminyltransferase 12
IPI00473603.1	0.632	3	Sjogren syndrome antigen B
IPI00754562.1	0.644	2	Isoform 1 of Membrane-associated phosphatidylinositol transfer protein 2
IPI00124590.3	0.669	6	CASP8-associated protein 2
IPI00319992.1	0.689	27	78 kDa glucose-regulated protein precursor
IPI00668028.2	0.691	4	nucleoporin 210 kDa-like
IPI00411016.1	0.697	3	Lacrimal androgen-binding protein epsilon
IPI00123639.1	0.699	12	Calreticulin precursor
IPI00352163.3	0.706	2	Fibronectin 1
IPI00163011.2	0.736	4	Thioredoxin domain-containing protein 5 precursor
IPI00652063.2	0.737	3	FAT tumor suppressor homolog 3 isoform 2
IPI00131533.1	0.761	2	Caspase-8 precursor
IPI00114342.1	0.782	2	Hexokinase-2
IPI00129796.2	0.790	2	POU domain, class 3, transcription factor 1
IPI004657896.3	0.795	3	Talin-1
IPI00667941.1	0.800	3	Inositol 1,4,5-trisphosphate 3-kinase B isoform 2
IPI00410989.2	0.804	4	ATP-binding cassette transporter sub-family A member 16
IPI00223699.4	0.811	4	Extracellular matrix protein FRAS1 precursor
IPI00113575.1	0.813	3	Kallikrein 1-related peptidase b16 precursor
IPI00348451.5	0.818	2	Caspase recruitment domain protein 12
IPI00658501.1	0.820	6	Microtubule-associated protein 1 A isoform 5

**Table 2:** A partial list of up-regulated SMG proteins in BM SOUP-treated NOD mice.

Accession Number	Ratio 131/130	# of Peptides	Protein Name
IPI00121550.1	1.202	9	Sodium/potassium-transporting ATPase subunit beta-1
IPI00128296.1	1.204	6	Creatine kinase, ubiquitous mitochondrial precursor
IPI00122958.1	1.205	2	Induced myeloid leukemia cell differentiation protein Mcl-1 homolog
IPI00663736.1	1.205	3	Similar to Ras GTPase-activating protein SynGAP
IPI00466650.1	1.209	3	Isoform 2 of Calcium/calmodulin-dependent 3′,5′-cyclic nucleotide phosphodiesterase 1C
IPI00123183.2	1.234	2	Aquaporin-1
IPI00129264.1	1.237	2	Vinexin
IPI00114982.1	1.247	2	Signal transducer and activator of transcription 5B (STAT5B)
IPI00274030.3	1.247	4	splicing factor, arginine/serine-rich 8 isoform 1
IPI00410905.1	1.250	2	Isoform 5 of Calcium-activated potassium channel alpha subunit 1
IPI00130116.1	1.303	2	Transient receptor potential cation channel subfamily M member 2
IPI00222042.1	1.328	3	Sorting nexin-14
IPI00622013.2	1.334	2	RAS-like family 11 member B
IPI00130920.1	1.363	2	Microtubule-associated protein 1B
IPI00400329.2	1.385	2	NF-kappa-B-repressing factor
IPI00380983.3	1.387	2	MRS2-like, magnesium homeostasis factor
IPI00315893.1	1.537	10	Alpha-amylase 1 precursor
IPI00453692.3	1.585	3	Nestin
IPI00227299.5	1.610	6	Vimentin
IPI00223523.1	1.980	3	Autoimmune regulator, isoform 3b
IPI00626585.2	2.301	2	Calcium-dependent secretion activator 2, isoform 5
IPI00131763.1	2.445	7	Parotid secretory protein precursor
IPI00122048.2	2.928	14	Sodium/potassium-transporting ATPase alpha-3 chain, Aquaporin pathway related

### Functional Pathway Analysis

DAVID/KEGG pathway analysis was performed on the differentially expressed proteins to evaluate which pathways were significantly represented in BM Soup treated SMG tissue cells. Four pathways were found to be significant (p≤0.01) including calcium signaling pathway, inositol phosphate metabolism, focal adhesion and extracellular matrix (ECM) - receptor interaction (**Table 3**). These four pathways play an important role in the differentiation and apoptosis of different cell types including stem cells.

**Table 3:** The KEGG pathways associated with BM Soup treatment as revealed by the DAVID analysis.

Functional pathway	Protein count	P value
Calcium signaling pathway	12	0.002
Inositol phosphate metabolism	6	0.004
Focal adhesion	11	0.006
ECM-receptor interaction	7	0.006

### qPCR Analysis of Gene Expression in the SMGs of BM Soup Treated NOD Mice

**Figure 3:**
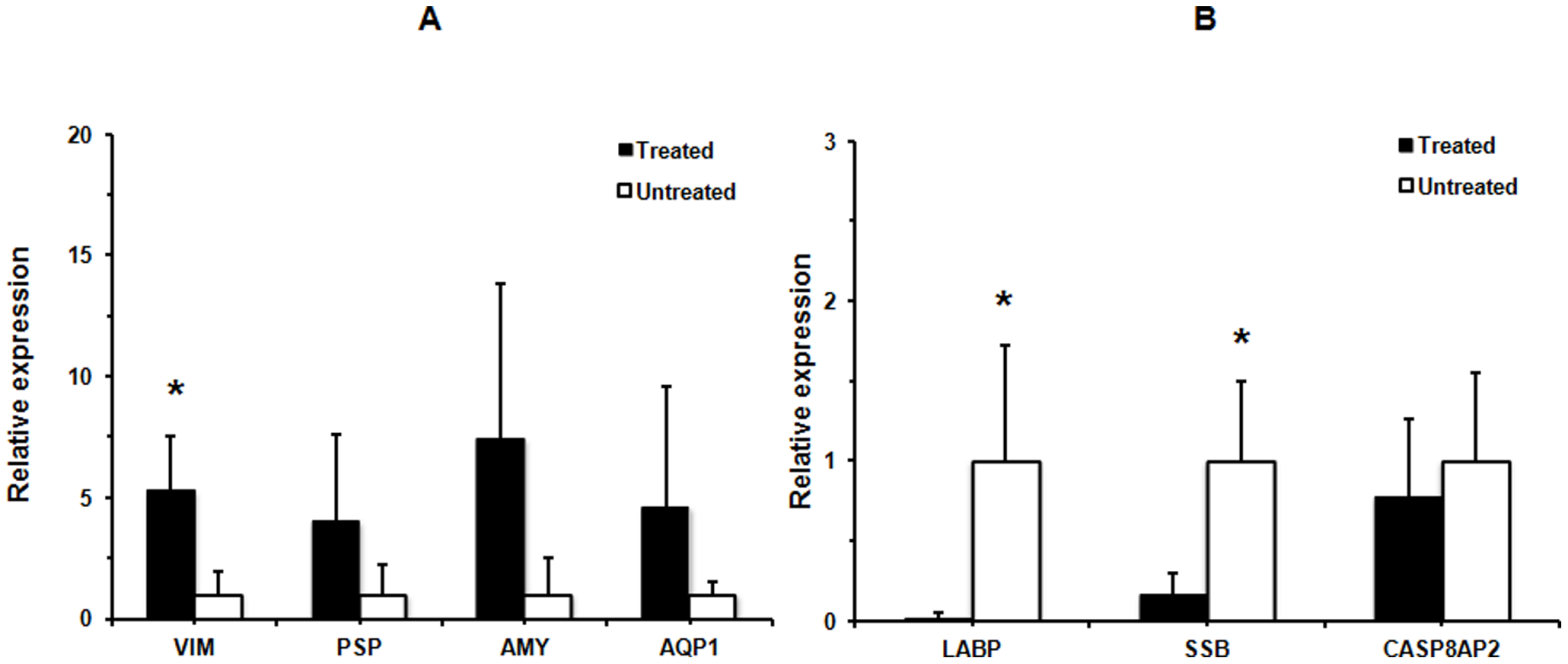
Relative gene expression of VIM, PSP, AMY, AQP1, LABP, SSB, and CASP8AP2 in the SMGs of the BM SOUP-treated NOD mouse group (n = 4) versus the untreated NOD mouse group (n = 4). The mRNA levels of VIM, LABP and SSB were found to be significantly altered in the treated NOD group (*p<0.05). Although the average mRNA levels were increased with 4-fold for PSP, 7.5-fold for AMY and 4.7-fold for AQP1 in treated NOD group, the differences were not statistically significant, which may be presumably due to limited sample size for each studied group.

Four up-regulated proteins in the treated NOD group, including alpha-amylase (AMY), vimentin (VIM), parotid secretory protein (PSP) and aquaporin 1 (AQP1), and three down-regulated proteins in the treated NOD group, including lacrimal androgen-binding protein (LABP), Sjogren’s syndrome antigen B (SSB) and caspase 8 associated protein 2 (CASP8AP2), were further tested at mRNA level with qPCR. As shown in **Fig. 3**, qRT-PCR analysis indicated that three out of seven genes (VIM, LABP and SSB) were significantly altered in treated NOD group versus untreated NOD group (p<0.05). Although the difference is not statistically significant presumably due to limited sample size, the average gene expression levels were increased around 4-fold for PSP, 7.5-fold for AMY and 4.7-fold for AQP1 in treated NOD group when compared to untreated NOD mouse group.

**Figure 4:**
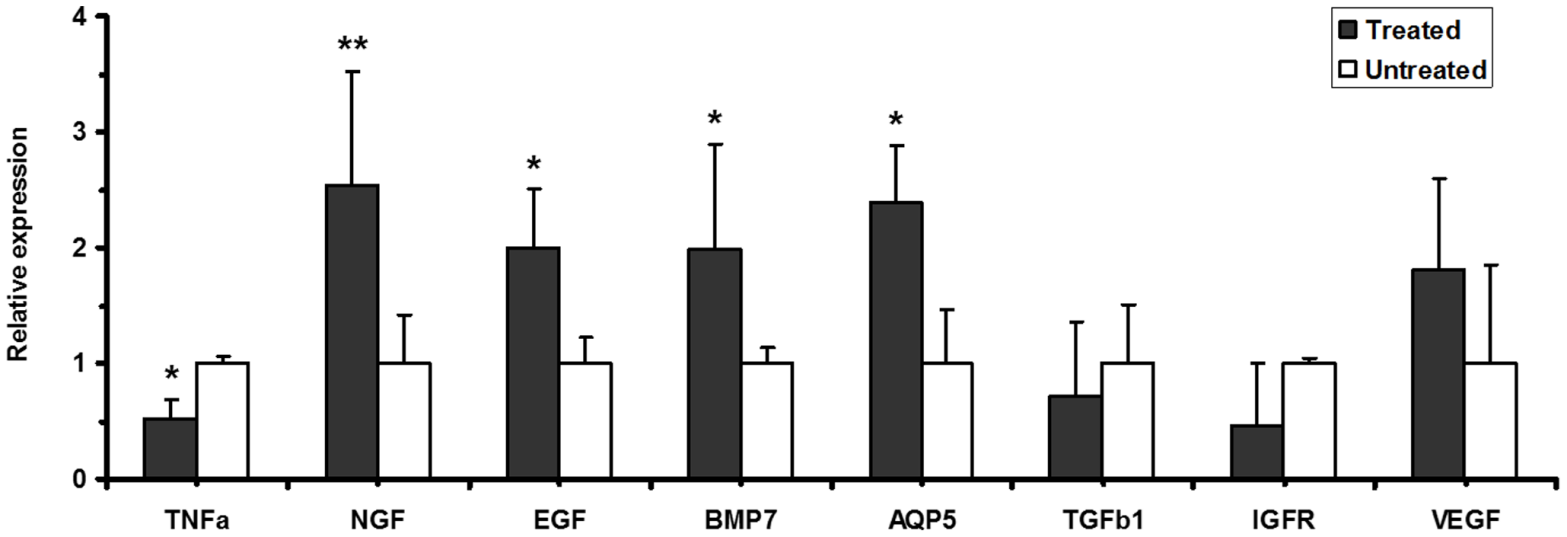
Relative gene expression of TNFa, TGFb1, IGFR, EGF, NGF, VEGF, BMP7 and AQP5 in the SMGs of the BM SOUP-treated NOD mice (n = 4) versus the untreated NOD mice (n = 4). The mRNA levels of TNFa, EGF, NGF, BMP7 and AQP5 were significantly altered in the treated NOD group (*p<0.05; **p<0.01).

BM Soup treatment also altered the expression of growth factors/cytokines in the SMGs of NOD mice. Growth factors and cytokines are relatively low abundant, therefore they were not readily detected with the MS-based proteomic approach. However, as shown in **Fig. 4**, qRT-PCR analysis indicated that epidermal growth factor (EGF) and nerve growth factor (NGF) were significantly up-regulated whereas tumor necrosis factor alpha (TNFa) was significantly down-regulated in the BM Soup-treated NOD group. Meanwhile, AQP5 (aquaporin 5) and BMP7 (bone morphogenetic protein 7), which is a member of the TGF superfamily, were also significantly up-regulated in the treated group. There were slight changes in the gene expression of VEGF (vascular endothelial growth factor) and transforming growth factor beta 1 (TGFb1) between the untreated and treated groups but the differences were not statistically significant.

## Discussion

One of the main findings in our study is that BM Soup treatment effectively improved the SFR of NOD mice. SFR directly reflects function of the glands and its decrease is one of the major clinical findings in patients with Sjogren’s syndrome (SS). Therefore, an improved SFR of the NOD mice indicated that BM Soup treatment was efficient in restoring salivary gland function. In addition, BM Soup treatment inhibited the lymphocytic infiltration of SGs in the NOD mice, as evidenced by significantly reduced focus scores in the treated NOD mice. Timing in testing experimental therapies in NOD mice is a critical factor. NOD mice develop Sjogren’s-like disease (SS-like) in three phases [32]. Phase 1 (from 0 to 8 wks of age) is independent of detectable autoimmunity. In phase 2 (8–16 wks; onset of disease), the autoimmunity believed to result from the acinar cell apoptosis leads to leukocytes infiltrating in the SGs. After 16 wks of age (Phase 3), loss of SG secretory function occurs (advanced disease). Our group has tested BM cell therapy in NOD at different phases of SS-like disease, such as at 8, 14, and 20 weeks of age [18], [33]–[35] . In our latest study [35], we injected a combined-therapy (consisting of immuno- and bone marrow cell-based therapy) during either the initial phase of SS-like (8-week old NOD), or at an advanced phase (20-week old NOD). We reported that BM cell therapy maintained salivary flow rates between 80–100% of pre-symptomatic levels when given at 8 weeks of age, while when the therapy was given at an advanced phase of SS-like (20 weeks and older), salivary flow rates improved, but were at best 50% of pre-symptomatic levels. Since the goal of this manuscript is to show proof-of-concept of a new therapy (BM Soup) against SS-like disease, we provided the therapy at the critical time point of 8 weeks of age to NOD to maximize our chances to detect a significant improvement in saliva flow rate.

At the molecular level, BM Soup treatment altered the protein expression of a number of genes, particularly those involved in the proliferation, inflammation, apoptosis as well as tissue repair and regeneration in the SGs of NOD mice (see summary in Table 4). Our studies showed that α-amylase was up-regulated at the protein level in the SMG cells of BM Soup-treated NOD mice when compared to untreated NOD mice. Although the difference in the gene expression of α-amylase was not statistically significant due to limited sample size in each studied groups, the mRNA level of α-amylase was increased at 7.5-fold in treated NOD group when compared to untreated NOD group. Baldini et al. found that there was a significant decrease of α-amylase in SS patients compared to healthy and non-SS sicca syndrome subjects [36]. Previous report also revealed that the decrease of amylase in SS is due to acinar parenchymal damage [28], and the expression of acinar-specific markers such as α-amylase and cystatin could help differentiate ductal cells into acinar cells [37]–[39]. The up-regulation of α-amylase in BM Soup-treated NOD mice suggests that the protein expression of salivary α-amylase in NOD mice was restored by BM Soup treatment. The elevated levels of salivary α-amylase may help differentiate ductal cells to acinar parenchymal cells.

**Table 4:** A list of potential target proteins of action by BM SOUP treatment.

Protein name	Current study	Previous study	References
**Inflammation-related**			
Kallikrein 1- related peptidase	Down-regulated in BM SOUP-treated NOD mice	Up-regulated in salivary glands of IQI/Jic mice	[52]
Calreticulin/Calmodulin	Down-regulated in BM SOUP-treated NOD mice	Up-regulated in salivary glands of IQI/Jic mice	[24], [25]
**SS biomarker**			
Sjogrens syndrome antigen B	Down-regulated in BM SOUP-treated NOD mice	Up-regulated in SS patients	[9], [63]
**Salivary gland biology**			
α-Amylase	Up-regulated in BM SOUP-treated NOD mice	Down-regulated in SS patients	[36]–[39]
Aquaporin-1	Up-regulated in BM SOUP-treated NOD mice	Down-regulated in SS patients	[40], [41], [43]
Aquaporin-5	Up-regulated in BM SOUP-treated NOD mice	Down-regulated in SS patients	[40], [41], [43]
Parotid secretory protein	Up-regulated in BM SOUP-treated NOD mice	Down-regulated in SS patients	[44], [45]
**Stem cell and development**			
Nestin	Up-regulated in BM SOUP-treated NOD mice	Up-regulated in repaired lacrimal glands	[47], [64]
Vimentin	Up-regulated in BM SOUP-treated NOD mice	Up-regulated in repaired lacrimal glands	[47], [64]
**Apoptosis**			
CASP8-associated protein 2	Down-regulated in BM SOUP-treated NOD mice	Up-regulated in SS	[49], [65]
Caspase-8	Down-regulated in BM SOUP-treated NOD mice	–	–
Caspase recruitment domainprotein 12	Down-regulated in BM SOUP-treated NOD mice	–	–

We also found that aquaporin 1 (AQP1) protein was up-regulated in the SMG cells of the NOD mice by the BM Soup treatment. Although the difference in the gene expression of AQP1 was not statistically significant due to limited sample size in the studied groups, the average level of AQP1 mRNA was increased at 4.7-fold in treated NOD group when compared to untreated NOD group. Aquaporins are water channel proteins that play an important functional role in salivary secretion. AQP1, AQP3, AQP4 and AQP5 are expressed and localized in major human salivary glands (parotid, SMG, sublingual) as well as labial glands [40], [41]. In fact, AQP1 is expressed in salivary gland myoepithelial cells. The presence of AQP1 in myoepithelial cells plays an important role in saliva secretion, since myoepithelial cells embrace acinar cells; the aquaporin might ensure the water flow into the basal aspect of the acinar epithelial cells [42]. In addition, the expression of AQP1 was significantly down-regulated in the salivary gland tissue cells of SS patients [43]. Similarly, as indicated by the qPCR analysis, the gene expression of AQP5 was significantly up-regulated in the BM Soup-treated NOD group versus untreated NOD group (p<0.05, Figure 4). Our results suggest that the BM Soup treatment elevates the expression of AQP1 and AQP5 in the SMG cells, which may contribute to the improved saliva flow rates in NOD mice.

BM Soup treatment also led to the up-regulation of parotid secretory protein (PSP, >2.4 fold increase) in the NOD mice, which is a secreted protein involved in binding and clearing various infectious agents [44], [45]. qPCR analysis also indicated a 4-fold increase of average PSP mRNA expression level in the treated NOD group although the difference was not statistically significant. Previous studies indicated that aberrant synthesis and processing of PSP in the SMGs of NOD-scid mice correlates to the time of appearance of lymphocytes in the parental NOD mice. It also correlates with a loss of acinar cells through time and an increase of a ductal cell population [44]. The elevated expression of PSP protein in BM Soup treated NOD mice may contribute to the decreased activity of infiltrating lymphocytes in the salivary glands.

Both vimentin and nestin were up-regulated at protein level in BM Soup-treated NOD mice versus untreated NOD mice. The mRNA expression level of vimentin was also significantly up-regulated in the treated NOD mouse group (p<0.05). Nestin is a stem cell marker expressed by many types of cells during development while vimentin is expressed in mesenchymal cells and involved organogenesis, wound healing and tumor invasion. In fact, vimentin appears as an important biomarker in epithelial-mesenchymal transition (EMT) which is responsible for generation of stem cells during the tissue repair [46]. In a previous study, You et al. found that vimentin and nestin were both up-regulated during repair of lacrimal glands in mice. Their results showed that there was a presence of a heterogeneous population of mesenchymal stem cells (MSCs) based on the expression of nestin and vimentin in the lacrimal glands [47]. Since MSCs are the major type of stem cells in bone marrow, our finding suggests that the factors present in BM Soup induced the expression of nestin and vimentin, which might stimulate the tissue regeneration and repair in the SMGs of NOD mice.

Proteomic analysis also revealed the down-regulation of Sjogren syndrome antigen B (SSB), caspase 8 (CASP8), CASP8-associated protein 2 (CASP8AP2), and kallikrein-1 related peptidase in BM Soup-treated NOD mice versus the untreated NOD mice. qPCR analysis further confirmed that the mRNA level of SSB was significantly down-regulated in the treated NOD mice. Caspases play an important role in the initiation and execution of the apoptosis process. They are synthesized as inactive zygogens that become activated by cleavage via upstream proteases and, once activated, lead to cell death via extrinsic or intrinsic pathways [48]. The extrinsic pathway involves the death receptor such as Fas and is initiated by the TNF-receptor family members, leading to the cleavage and activation of initiator caspase-8 [48]. Indeed, increased apoptosis of salivary gland epithelium was observed in SS patients, with elevated expression of Fas and FasL in glandular ductal and acinar cells [49]. These results imply that BM Soup treatment inhibits the expression of caspase-8 and apoptosis of SMG cells in the NOD mice.

Sjogren’s syndrome antigen B (SSB) is an autoantigen associated with autoimmune disorders. In fact, serum anti-SSB/La autoantibody serves as a commonly used marker for diagnosis of SS. Previously we found that salivary anti-SSB/La level was significantly higher in primary SS patients compared with systemic lupus erythematosus (SLE) and healthy control individuals [9]. In the present study, the BM Soup treatment down-regulated the expression of SSB autoantigen in the SMG cells, which suggests a decreased autoimmune activity in the BM Soup-treated NOD mice. Kallikreins are a group of serine proteases classified into two categories, plasma kallikreins (blood plasma) and tissue kallikreins (glandular organs) [50]. Kallikrein cascade play an important role in the initiation and maintenance of inflammatory responses [51]. In fact, both kallikrein-1 and kallikrein-13 were found to be up-regulated in the salivary glands of IQI/Jic mice [52]. Our study seems to indicate that the BM Soup treatment decreased the activity of kallikrein 1 in the NOD mice.

The KEGG pathway analysis of proteomic data indicates that some of the differentially expressed proteins are associated with calcium signaling, inositol phosphate metabolism and focal adhesion pathways. Calcium plays an important role in biological signaling, protein secretion, exocytosis, and muscle contraction [53], [54]. Oscillations in Ca^2+^, in response to Ca^2+^ mobilizing stimuli, are observed in many non-excitable cells, such as pancreatic acinar cells, oocytes, liver cell, and fibroblast [55]. Calmodulin and calreticulin are calcium binding proteins which mediate the biological effects of Ca^2+^ [56]. These proteins were found to be up-regulated in salivary gland cells of SS patients which might be due to the interaction with autoantigen La/SSB [25], [26]. The over-expression of calreticulin, in fact, caused a decrease in the rate of Ca^2+^ mobilization from the internal stores [57]. We found that calreticulin expression in SMG cells was down-regulated by BM Soup treatment, which may restore the Ca^2+^ signaling and flow in the SMG cells of NOD mice. Meanwhile, we found inositol 1,4,5- trisphosphate 3-kinase B (IP3K) and membrane-associated phosphatidylinositol transfer protein 2 were down-regulated in BM Soup-treated NOD mice. Both proteins are involved in inositol phosphate metabolism. Inositol 1,4,5-trisphosphate (IP_3_) is a secondary messenger and responsible for the regulation of Ca^2+^ activation/chloride efflux and also has a role in inositol phosphate metabolism pathway [58]. The phosphatases and kinases present in the metabolic cascade generate a range of inositol phosphate derivatives such as IP_3_ and phosphatidylinositol [59]. They may be associated with the homeostasis of calcium in the SMG tissue cells of BM Soup-treated NOD mice.

Talin-1, actin and extracellular matrix protein FRAS1 precursor were also down-regulated in BM Soup-treated mice. These proteins are associated with the focal adhesion pathway. Focal adhesions (FAs) are complex plasma membrane-associated macromolecular assemblies that serve to physically connect the actin cytoskeleton to integrins that engage with surrounding extracellular matrix (ECM) [60]. FA-related signaling networks dynamically modulate the strength of the linkage between integrin and actin and control the organization of the actin cytoskeleton [60]. In a previous study, the expression of FA proteins was found to be lower in adult salivary acinar population when compared with embryonic glandular tissue [61]. In SS, focal adhesions may be constantly assembled as the motile lymphocytes establish new contacts at the leading edge following cellular signals to damaged biological tissue. Our study seems to suggest that BM Soup treatment inhibits the FAs in the SMGs of the NOD mice.

Lastly but importantly, BM Soup treatment also altered the expression of growth factors and cytokines in the SMGs of NOD mice. Due to their low abundance, these molecules are not readily detected with the MS-based proteomic approach. As shown in **Fig. 4**, qRT-PCR analysis indicated that epidermal growth factor (EGF), nerve growth factor (NGF), and BMP7 (bone morphogenetic protein 7) were up-regulated whereas tumor necrosis factor alpha (TNFa) was down-regulated in the SMGs of the NOD mice treated by BM Soup. The reduced expression of TNFa suggests that BM Soup treatment inhibited the inflammation in the SMGs of the NOD mice. However, the over-expression of growth factors EGF, NGF and BMP7 implies that BM Soup treatment may repair the damaged SGs in NOD mice through the regulation of these growth factors involved in SG development/maintenance. In fact, direct injections or vector-mediated transfer of growth factors (such as FGF2, IGF, KGF or VEGF) without cell transplants have been reported partially effective in repairing irradiated SGs in mice [22].

Taken together, these results suggest that BM Soup (cell extract) is a novel and promising strategy to SS treatment because animal model studies have shown that transplants with BM Soup can functionally restore the damaged salivary glands in SS mouse models. Through the analysis of protein/gene alterations in the SMG tissues of the BM Soup-treated NOD mice, we have found that BM Soup treatment might be effective due to inhibiting apoptosis, focal adhesion and inflammation whereas promoting the development, regeneration and differentiation of SG cells. These findings provide important insights on the potential mechanisms underlying the BM Soup treatment for functional restoration of damaged SGs in NOD mice. However, there was discrepancy between proteomics and qPCR analysis for a few genes, which might result from a limited sample size in the studied animal model groups. Although it is known that protein and mRNA expression levels may not be well correlated, additional studies are certainly warranted to further confirm the identified target genes and their related signaling pathways that are responsible for the BM Soup treatment.

## Materials and Methods

### Animals and BM Soup Preparation

All procedures with animal studies were approved by the Institutional Animal Care and Use Committee (IACUC) at the McGill University (Approved protocol #5330, www.animalcare.mcgill.ca). Seven week-old female NOD mice from Taconic Farms (Germantown, NY) were housed untouched for a one-week acclimatization period in the Genome Quebec Animal Facility at McGill University. Then they were randomized into two groups: a) BM Soup treatment (n = 6) and b) no treatment (n = 6) Female NOD mice from the “BM Soup” group were injected via the lateral tail vein using an approved restrainer and a 29G syringe, twice weekly for 2 consecutive weeks, with the BM Soup extracted from male donor MHC class Ι-matched bone marrow cells harvested from aged-matched CByF1B6F1/J mice (Jackson Laboratory, NY). Donor mice were sacrificed and then the muscles were immediately removed and the bones were exposed. Briefly BM Soup was obtained by flushing BMDCs from tibias and femurs with cold PBS. The suspension was then strained through a 40-µm filter and washed again with PBS. Cell concentration was adjusted to 10^7^ cells/100 µl PBS. BM Soup was prepared as described by Yeghiazarians et al [62], with modifications, by subjecting BMDCs to three cycles of freeze-thaw on a container with dry ice placed in a −80C freezer and thawing in a 37C bath, followed by microcentrifugation at 13,500 g at 4C for 30 min to remove insoluble materials. Protein concentration of BM Soup was ∼1.3 µg/µl (from 10^7^ cells). Non-treated NOD mice (i.e. the “Control group”, n = 6), were expected to develop diabetes during the course of the follow-up period, and therefore any mouse with a glucose level above 250 mg/dL received daily injections of insulin to control their blood sugar levels.

At 20 weeks of age (i.e. 12 weeks after BM Soup therapy), blood was taken from NOD mice using cardiac puncture method and then the mice were sacrificed. The SMG glands were immediately removed from the mice and frozen at −80°C. Due to gland dissection and sample preparation issues, we only had high quality RNA and protein samples from 5 treated NOD mouse glands and 4 untreated NOD mouse glands. For the comparative qPCR analysis, we used an equal sample size of n = 4 per group.

Euthanasia of mice was performed using carbon dioxide asphyxiation. In order to minimize stress, the mice were sacrificed in their home cage (7.25″×11.5″×5″) with a maximum of five adult mice and no mice were pooled from different cages. The regulator of carbon dioxide was set as 2 L/min for standard mouse cage (7.25″×11.5″×5″). Once the mice became unconscious, the flow rate was increased to minimize the time of death and maintained until the animals stopped breathing. Afterwards, the regulator was closed and the mice were left in contact with carbon dioxide for an additional 2 minutes. To confirm death, the mice was checked for the following signs: no rising and falling of chest, no palpable heart beat, poor mucous membrane color, no response to toe pinch, color change or opacity in eyes.

### Measurements of Salivary Function

Salivary flow rate (SFR) was obtained by using inhalation anesthetic. Anesthesia was accomplished using 5% Isoflurane, 5% Halothane, and 0.5–1 L/min oxygen for induction and 1% Isoflurane, 1% Halothane and 0.5 L/min Oxygen for maintenance. 0.5% L/min Oxygen was used during the recovery period. Whole saliva was collected after stimulation of secretion using 0.5 mg pilocarpine/kg body weight administered subcutaneously. Saliva was obtained from the oral cavity by micropipette, placed into pre-weighed 0.5-ml microcentrifuge tubes. Saliva was collected for a 10-minute period and its volume determined gravimetrically. SFR was determined at week 10, week 16, and week 20 of age. Linear Mixed Models and ANOVA analysis (Tukey’s Post Hoc test) were used to determine statistical differences (P<0.05) between the mouse groups. Mice between and within the groups were compared at different time points using the SPSS version 17 (IBM, USA).

### Salivary Tissue Analysis

Focus score was performed as previously described [18]. Half of a submandibular gland per mouse was fixed in 10% formalin and embedded in paraffin. Sections were cut at 5–8 µm thick and subsequently stained with hematoxylin and eosin (H&E). A score of 1 is a foci (aggregate) of at least 50 inflammatory cells per 4 mm^2^ of salivary tissue.

### Tandem Mass Tagging

The SMG tissues of NOD mice were lysed in 8 M urea with a homogenizer. Then, the samples were centrifuged at 15,000 rpm for 5 minutes at 4°C, the supernatants were removed and the levels of proteins were quantified by the Bradford method. Afterwards, the lysed samples were labeled with TMT Mass Tagging Kit (TMT 6plex, Thermo Scientific). In brief, the SMG tissue samples were precipitated overnight at −20°C with cold ethanol. Following centrifugation at 4°C, speed 13500 RPM for 15 minutes. After removing amine-based buffers and thiol reagents, samples were reduced, alkylated and digested overnight. The control tissue sample was labeled with reagent 130 whereas the BM Soup-treated sample was labeled with reagent 131. Afterwards, we combined the labeled samples and performed the strong-cation exchange using the VIVAPURE S mini H filter (Sartorius stedim Biotech). The filter was pre-wet with sodium acetate (25 mM) and centrifuged 2000×g for 10 minutes. The combined samples were added to the filter and centrifuged 2000×g for 10 minutes at room temperature. The first filtrated sample was collected and eight subsequent step elution (2.5 mM, 5 mM, 10 mM, 20 mM, 50 mM, 100 mM, 250 mM and 1 M) were performed and fractions were vacuum dried. The dried samples were resuspended with 0.1% formic acid prior to LC-MS/MS.

### 2-D LC with MS/MS

Fractionated peptide samples were loaded on an Agilent nanotrap column (Santa Clara, CA) and washed for 10 minutes at 6 µl/min. Chromatography was performed using Eksigent 2D-LC nanoflow system operating at 400 nl/min and a 90 minute gradient. Separation was performed on a Microm 100×0.1 mm C18AQ column (200A, 3 um) using solvent A (0.1% Formic Acid) and solvent B (99.9% ACN, 0.1% formic Acid) over 90 minute gradient: (0–30% B (60 min), 35–80% B (10 min), 80% B (5 min), and then the column was re-equilibrated). Data Dependent LC-MS-MS was performed using an Orbitrap LTQ XL mass spectrometer (Thermo Fisher, San Jose, CA) with the MS scan performed in the Orbitrap analyzer using 2 microscans of maximum time 50 ms and an automatic gain control of 1E5. The top 3 ions of intensity greater than 5000 (excluding single charge states) were selected for MS-MS. In each cycle, a MS-MS fragmentation was generated using subsequent CID (collision energy 35) and HCD (collision energy 42) scans performed in the LTQ Iontrap and Orbitrap cell, respectively, which were then combined in data processing to obtain quantitative and qualitative data.

### Bioinformatics Analysis

Database searching of tandem MS data was performed using the Proteome Discoverer 1.2 (Thermo Fisher Scientific) and a workflow created by the purpose of analysis of the raw data obtained from LC-MS/MS. In brief, the files obtain from mass spectrometry were *.RAW file and all data were loaded into the *Spectrum Files*. The parameters for *Spectrum Selector* were set as default. Next, the *Reporter Ions Quantifier* was set for TMT 6plex (using 130 and 131). The data searching parameters were set as the static modifications were N-terminus TMT6plex (+229.163 Da), peptide-C terminus none and Carbamidomethyl (Rev1/+57 Da). Proteins with high confidence were selected according to standard identification criteria.

Gene Ontology and functional pathway analysis were performed with the online informatics analysis tool - Data for Annotation, Visualization and Integrated Discovery (DAVID). International Proteome Index (IPI) accession numbers for quantified proteins were converted into UniGene IDs and then uploaded onto the DAVID for analysis.

### Quantitative Real-Time PCR (qRT-PCR)

Gene expression analyses were performed using an Applied BioSystems quantitative Real Time PCR (qRT-PCR) system (model 7500). Salivary tissues were preserved in RNAlater solution (Qiagen, Germantown, MD, USA). The extracted RNA integrity number (RIN) was verified to be over 6 with an Angilent Bioanalyzer 2100 (Agilent Technologies, Santa Clara, CA, USA). The first-strand cDNA synthesis was performed by using Thermoscript RT-PCR system (Invitrogen). qRT-PCR was done using 1 µg RNA per sample and TaqMan Universal Master Mix (Applied Biosystems). The probes and primers sequences were: Tumor necrosis factor alpha (*TNF-α*; Assay ID: Mm00443258), Epidermal Growth Factor (EGF; assay ID: Mm 00438696), Transforming Growth Factor-β (TGF-β; assay ID: Mm01178820), Vascular Endothelial Growth Factor (VEGF; assay ID Mm01281449), Aquaporin 5 (AQP5; Assay ID: Mm 437578), insulin like growth factor receptor 1 (IGF-IR; Assay ID: Mm00802841), Bone Morphogenetic Protein 7 (BMP7; assay ID: Mm00432102), Nerve Growth Factor (NGF; assay ID: Mm00443039). Glyceralhyde-3-phophate dehydrogenase (GAPDH; Mm99999915-g1) and beta-actin (ACTB, Mm00607939-s1) were used as endogenous reference genes. The primers for the rest of genes including vimentin (VIM), parotid secretory protein (PSP), alpha amylase (AMY), aquaporin 1 (AQP1), lacrimal androgen-binding protein (LABP), Sjogren’s syndrome antigen B (SSB), and caspase 8 associated protein 2 (CASP8AP2), are shown in Table S2. The experiments were run at 50°C for 2 min, 95°C for 10 min, and then for 40 cycles at 95°C for 15 s, and 60°C for 1 min.

As for qPCR data analysis, the expression levels of genes in treated NOD mouse samples were plotted in relative to the expression levels of genes in untreated NOD mouse samples. Statistical analysis was performed with the Mann–Whitney–Wilcoxon test (a.k.a., Wilcoxon rank-sum test) in the MedCalc statistical software (MedCalc Software, Belgium) and p<0.05 was considered as statistically significant.

## Supporting Information

Table S1 **A list of quantified SMG proteins between BM Soup-treated and untreated NOD mice.**
(XLSX)

Table S2 **Primer sequences for qPCR analysis.**
(DOCX)
