# Supplementary material for: Quantitative Analysis of Protein and Gene Expression in Salivary Glands of Sjogren’s-Like Disease NOD Mice Treated by Bone Marrow Soup
Source: PLoS One. 2014 Jan 29;9(1):e87158. doi: 10.1371/journal.pone.0087158 (PMC3906116; doi:10.1371/journal.pone.0087158)
Supplement: Table S2 — Primer sequences for qPCR analysis. (DOCX) [file pone.0087158.s002.docx]

**Supplemental Table 2. Primer sequences for qPCR analysis**

| Gene | Forward Sequence (5’ to 3’) | | Reverse Sequence (5’ to 3’) |
| --- | --- | --- | --- |
| Amylase, alpha | CTGGGGGAGCATCCATCTTG | CCAGTCATTGCCACAAGTGC | |
| Aquaporin 1 | ACCTGCTGGCGATTGACTAC | TTTGGGCTTCATCTCCACCC | |
| Parotid secretory component protein | GCTGTCTTCCAACGGCAATG | ATGTCGACCGTCTTGCCAAT | |
| Caspase 8 associated protein 2 | GGAAGAGACCTGTGGTGGTG | GGTGCAAGATGGCTCTCACT | |
| Lacrimal androgen-binding protein | CTGGGCTTCCAGAGAACGGA | AACCCAATCCTGTTTCCAGAGA | |
| Vimentin | AGCAGTATGAAAGCGTGGCT | CTCCAGGGACTCGTTAGTGC | |
| Sjogren’s syndrome antigen B | TCTTTAAGCGCGTTTGGCTG | AAGATTCGGATCCCACGGTC | |
